# Cross-cultural adaptation and validation of the Turkish version of the arthritis self-efficacy scale-8 (ASES-8) in patients with knee osteoarthritis

**DOI:** 10.1097/MD.0000000000045599

**Published:** 2025-11-07

**Authors:** Mehmet Serkan Kiliçoğlu, Delal Öztürk, Buğra İnce, Ozan Volkan Yurdakul, Teoman Aydin

**Affiliations:** a Department of Physical Medicine and Rehabilitation, Bezmialem Vakif University, Faculty of Medicine, Fatih-Istanbul, Turkey; b Department of Physical Medicine and Rehabilitation, Kütahya City Hospital, Merkez-Kütahya, Turkey; c Department of Physical Medicine and Rehabilitation, University of Health Sciences Izmir Faculty of Medicine, İzmir City Hospital, Bayrakli-Izmir, Turkey.

**Keywords:** cross-cultural comparison, osteoarthritis of knee, patient reported outcome measures, self-efficacy, validity

## Abstract

Self-efficacy plays a key role in managing chronic conditions such as knee osteoarthritis (KOA), and reliable tools are needed to assess it in different languages and cultures. The arthritis self-efficacy scale-8 (ASES-8) is a widely used measure, but a validated Turkish version is not yet available. This study aimed to develop a Turkish version of the ASES-8 and evaluate its validity and reliability. The ASES-8 questionnaire was translated into Turkish following a standardized cross-cultural adaptation process. Initially, the clarity and comprehensibility of the Turkish version were tested in a sample of 10 patients with knee osteoarthritis (KOA). Subsequently, the final version was administered to an additional 80 KOA patients. All participants completed the questionnaire again 7 days later to assess test–retest reliability. Floor or ceiling effects were considered present if more than 15% of participants obtained the lowest (1) or highest (10) possible score on the scale. Construct validity was assessed in accordance with COSMIN guidelines by testing predefined a priori hypotheses regarding correlations between the ASES-8 and related measures, and structural validity through confirmatory factor analysis, testing a 1-factor (unidimensional) model. Reliability was evaluated through internal consistency (Cronbach’s α), test–retest reliability (intraclass correlation coefficient), and measurement error (SEMagreement and individual-level smallest detectable change). Confirmatory factor analysis supported the 1-factor structure, showing excellent fit indices (using the relative chi-square index, the goodness-of-fit index, the Tucker–Lewis index, and the normed fit index). The internal consistency was excellent, with a Cronbach’s α of 0.968. The test–retest reliability demonstrated strong stability for both the total scores and the individual items. Hypothesis testing for construct validity was established through strong positive correlations with the pain self-efficacy questionnaire and strong negative correlations with the Tampascale of kinesiophobia; the pain catastrophizing scale; the Western Ontario and McMaster Universities Osteoarthritis Index; and the depression, anxiety, and stress subscales of the depression anxiety and stress scale-42 (*r* = −0.80 to 0.91). No ceiling effect and minimal floor effect were observed at the total score level. The Turkish version of the ASES-8 has demonstrated evidence of validity and reliability for assessing self-efficacy in patients with knee osteoarthritis.

## 1. Introduction

Self-efficacy is a psychosocial variable that describes an individual’s confidence in their ability to perform a specific task or tasks.^[1]^ The concept is more closely related to the belief in one’s abilities than to their actual capabilities. Self-efficacy is often considered in knee osteoarthritis (KOA) management interventions because of its established effects on both overall health and treatment outcomes.^[2]^ It is regarded as a modifiable characteristic.^[2]^

KOA is one of the most common musculoskeletal disorders worldwide. It can have a considerable impact on an individual’s quality of life.^[3]^ Symptoms such as mobility limitations, chronic pain, and joint stiffness can restrict the ability to perform daily activities, potentially leading to social isolation.^[4]^ In individuals with KOA, a sense of self-efficacy can enhance both their ability to manage disease symptoms and their participation in physical activities.^[2]^ Among patients with KOA, those with high levels of self-efficacy participate more actively in rehabilitation programs and are more likely to achieve treatment goals.^[5]^ In recent years, various self-efficacy instruments have been developed to assess the self-efficacy of individuals with KOA.^[6–9]^ These instruments provide valuable insights into patients’ perceptions of their condition and degree of disability. They can be used in the development of strategies to increase self-efficacy levels and, in clinical practice, to optimize patients’ responses to treatment. Self-efficacy is increasingly recognized as an important determinant of health outcomes in patients with KOA,^[5–8]^ and understanding its role may help guide more effective clinical and public health interventions.

The arthritis self-efficacy scale (ASES) is widely used as a instrument of self-efficacy perceptions related to pain management, physical functions, and other disease symptoms.^[8]^ Compared with other self-efficacy assessment tools, the ASES has a longer history and is more applicable to KOA.^[2]^ Its validity and reliability have been demonstrated in several previous studies.^[10–12]^ The ASES was developed as part of the Stanford Arthritis Self-Management Study to assess self-efficacy and measure changes resulting from health education interventions.^[11]^

Originally, the scale originally comprised 20 items contributing to 3 subscales that assess perceptions of physical function, pain, and other symptoms. The shorter more practical 8 item ASES-8, was subsequently developed. This version includes 2 items from the ASES pain subscale, 4 items from the other symptoms subscale, and 2 new items addressing fatigue and pain prevention in daily activities. The items are questions, and the responses are ratings on a scale of 1 (very uncertain) to 10 (very certain), reflecting the patient’s level of confidence in their abilities.^[13]^

Although the ASES-8 has been validated and proven reliable in various languages, to the best of our knowledge, it has not yet been translated into Turkish and culturally adapted for Turkish patients with KOA.^[14–20]^ The adaptation and translation of patient reported outcome measures across different languages and cultures require specific methodologies to ensure proper linguistic translation and maintain the content validity of the measurement tool.^[21,22]^ Researchers aiming to develop effective and culturally appropriate interventions for Turkish-speaking KOA patients require valid, reliable, and culturally sensitive tools. However, to the best of our knowledge, a validated Turkish version of the ASES-8 does not currently exist.

## 2. Materials and methods

### 2.1. Study design

This prospective study aimed to develop a cross-cultural adaptation of the ASES-8 for patients with KOA and evaluate its construct validity and reliability. This study is reported in accordance with the CONSensus-based Standards for the Selection of Health Status Measurement Instruments (COSMIN).^[23,24]^ In accordance with the journal’s submission requirements, the STROBE checklist has also been completed and is submitted herewith.

### 2.2. Sample size

According to Watson and Thompson,^[25]^ suggest a sample size between 5 and 10 times the number of items on the scale. Based on this rule, a total of 80 participants were included.

### 2.3. Ethical approval

The study was approved by the Bezmialem Vakif University Ethics Committee (No. 2023/211; see Supplementary Digital Content 6, Supplemental Digital Content, https://links.lww.com/MD/Q528) and conducted in accordance with the tenets of the 1964 Declaration of Helsinki and its later revisions. Written informed consent was obtained from all participants prior to their enrollment.

### 2.4. Translation and cross-cultural adaptation

The translation and cross-cultural adaptation process was conducted in line with internationally accepted guidelines for adapting measurement tools used to assess health conditions.^[21,22]^ Official permission for the translation of the scale was obtained via email from the first author of the original scale, Prof Dr Kate Lorig (see Supplementary Digital Content 1, Supplemental Digital Content, https://links.lww.com/MD/Q528). Two bilingual rehabilitation specialists (MSK and DO) translated the scale items, response categories, and instructions into Turkish. Two independent bilingual language experts, both proficient in Turkish and English and experienced in academic translation and unaware of the original translation, subsequently back-translated the scale from Turkish into English. The experts involved in the translation process discussed and resolved any inconsistencies before finalizing the Turkish version of the ASES-8 (Supplementary Digital Contents 7 and 8, Supplemental Digital Content, https://links.lww.com/MD/Q528).

Face validity of the scale was assessed through cognitive interviews with 10 Turkish KOA patients of varying educational levels, ages, and disease durations. Participants evaluated the comprehensibility, appropriateness, and clarity of the scale items, and the feedback received was used to make linguistic adjustments. Once consensus was reached, the Turkish version was finalized. In addition, a feasibility pilot study was conducted with a different group of 10 patients prior to the main study to evaluate the practicality of administering the questionnaires. The average completion time was approximately 10 minutes, and participants reported that the instruments were practical and suitable for clinical use. These findings supported the feasibility of the data collection procedure and the decision to proceed with the full validation study.

### 2.5. Participants and procedures

Between April 15, 2025, and May 1, 2025, patients attending the researchers’ outpatient clinic were recruited as study participants by being directly invited to participate if they met the eligibility criteria. The inclusion criteria were being aged 18 years or older, having a diagnosis of knee osteoarthritis (KOA) according to the American College of Rheumatology criteria, having sufficient language skills to understand and complete the scale, and providing informed consent to participate. The exclusion criteria were health conditions unsuitable for a self-management program, behavioral or cognitive issues that could hinder the assessment process, and unstable angina or uncontrolled heart failure.

Data collection sessions were conducted on the same day as each participant’s routine medical follow-up appointment at the outpatient clinic and were repeated 7 days later. The administration was paper-based. The questionnaires were completed after the medical consultation, in the examination room, under the supervision of the researchers. Three researchers were responsible for data collection. During questionnaire distribution, the researchers answered participants’ questions but refrained from providing any guidance in order to maintain the impartiality of the responses. The same researchers used the same procedures, in the same room and under identical conditions, at both baseline and 7-day follow-up assessments. For participants with low literacy or those unable to read and write, the questionnaires were read-aloud by the researchers in the outpatient clinic room following a standardized protocol, and responses were recorded without any prompting or guidance to ensure consistency and impartiality. All participants completed both the initial and follow-up assessments, with a nonresponse rate of 0%.

All questionnaires used in our study were reviewed by all researchers, and hypothesis testing was conducted based on this evaluation. According to our predefined hypotheses, the construct validity of the Turkish ASES-8 was evaluated based on correlations with related instruments. A priori hypotheses regarding expected correlations were developed based on existing literature, the conceptual relevance of the comparator instruments, and expert discussions within the study team, which included clinicians and researchers with experience in musculoskeletal rehabilitation and health measurement. Specifically: A strong positive correlation (*r* = 0.60–0.80) was expected between the ASES-8 and the pain self-efficacy questionnaire (PSEQ), as both scales assess patients’ confidence in managing daily activities despite pain. Moderate negative correlations (*r* = –0.40 to –0.60) were anticipated between the ASES-8 and the depression, anxiety, and stress subscales of the depression anxiety stress scale (DASS-42), based on the rationale that higher self-efficacy is typically associated with fewer psychological symptoms. A strong negative correlation (*r* = –0.60 to –0.80) was also hypothesized with the Tampa scale for kinesiophobia (TSK), as higher self-efficacy is generally strongly associated with lower fear of movement or reinjury.^[26]^ Finally, a strong negative correlation (*r* = –0.60 to –0.80) was expected with the total score of the Western Ontario and McMaster Universities Osteoarthritis Index (WOMAC), given that better self-efficacy is strongly linked to reduced symptom burden and improved physical function in individuals with knee osteoarthritis.

### 2.6. Measurements

All participants completed the following battery of questionnaires. At the second data collection session, which was conducted 7 days later, only the Turkish ASES-8 was administered. All comparator instruments used in this study have validated Turkish versions and permission letters were obtained from the original developers (see Supplementary Digital Contents 2–5, Supplemental Digital Content, https://links.lww.com/MD/Q528).

### 2.7. Demographic data

A patient demographics questionnaire was administered at the same time as the Turkish ASES-8 to obtain sociodemographic and clinical data from the participants. The following information was collected: age, sex, height, weight, body mass index, smoking status, education level, disease duration, and treatments received.

### 2.8. Arthritis self-efficacy scale-8 (ASES-8)

The Turkish version of the ASES-8 was used to assess participants’ arthritis-related self-efficacy. The scale consists of eight items, each rated from 1 (very uncertain) to 10 (very certain). A total score is obtained by calculating the mean of all item scores; no subscale scoring is used.^[8]^

### 2.9. Pain catastrophizing scale (PCS)

The pain catastrophizing scale (PCS) consists of 13 items rated on a 5-point Likert scale ranging from 0 (“not at all”) to 4 (“all the time”). It includes 3 subscales: rumination, magnification, and helplessness. Higher scores indicate greater levels of pain catastrophizing.

The PCS has demonstrated reliability and validity in musculoskeletal populations, including those with osteoarthritis.^[27,28]^

### 2.10. Pain self-efficacy questionnaire (PSEQ)

The PSEQ is a 10-item scale designed to assess individuals’ confidence in performing daily activities despite chronic pain. Each item is rated on a 7-point Likert scale from 0 (“not at all confident”) to 6 (“completely confident”). Higher scores reflect greater self-efficacy in managing functional tasks.

The PSEQ has been shown to be valid and reliable in patients with chronic musculoskeletal pain, including osteoarthritis.^[9,29]^

### 2.11. Depression anxiety stress scale (DASS)

The DASS-42 includes 42 items rated on a 4-point Likert scale ranging from 0 (“did not apply to me at all”) to 3 (“applied to me very much or most of the time”). It contains 3 subscales – depression, anxiety, and stress – each with 14 items. Higher scores indicate greater severity of symptoms.

The DASS-42 has demonstrated strong psychometric properties in clinical populations.^[30]^

### 2.12. Tampa scale of kinesiophobia (TSK)

The TSK consists of 17 items scored on a 4-point Likert scale (1 = strongly disagree to 4 = strongly agree). Four items are reverse-coded (items 4, 8, 12, 16). Higher scores indicate increased fear of movement or (re)injury.

Scores above 37 suggest clinically relevant kinesiophobia.

The TSK has shown reliability and validity in musculoskeletal populations, including individuals with osteoarthritis.^[31,32]^

### 2.13. Western Ontario and McMaster Universities Osteoarthritis Index (WOMAC)

The WOMAC is a 24-item questionnaire designed to assess pain (5 items), stiffness (2 items), and physical function (17 items) in patients with knee and hip osteoarthritis. Items are rated using a Likert scale, with total scores ranging from 0 to 96. Higher scores indicate more severe symptoms and functional limitations.

The WOMAC has been validated in patients with knee osteoarthritis.^[33,34]^

Scores of all comparator instruments were used as originally scored. No transformation was applied to align their directionality with the ASES-8; therefore, both positive and negative correlations were expected and reported accordingly.

### 2.14. Missing data

No missing responses were observed for the Turkish ASES-8 or other administered scales.

### 2.15. Statistical analysis

SPSS for Windows, v. 26.0 (IBM Corp., Armonk), was used to perform all the statistical analyses. Continuous variables are reported as the means (standard deviations [SDs]) and medians (interquartile ranges [IQRs]) for normally and nonnormally distributed variables, respectively. Frequencies and percentages are provided for categorical variables. The item response distributions were assessed based on the skewness and kurtosis values of each item. These values were divided by their respective standard errors to calculate *z*-scores, using the ±2.58 threshold as a reference, which corresponds to a statistical significance level of 0.01. In addition, histogram plots were examined to visually inspect potential abnormalities in the distribution. Floor and ceiling effects were considered present if more than 15% of respondents selected the lowest (1) or highest (10) possible score.^[35]^

Construct validity was evaluated by testing the aforementioned a priori hypotheses. The conclusions were judged against the prespecified directions and magnitude thresholds described earlier, rather than p-values alone. The relationships between the Turkish ASES-8 score and the PSEQ, DASS, TSK, PCS, and WOMAC scores were evaluated via Pearson’s and Spearman’s correlation analyses. Correlation coefficients of 0.3 to 0.5 were considered low, values of 0.5 to 0.7 were considered moderate, and values of 0.7 to 0.9 were considered strong correlations.^[36–38]^

Structural validity was assessed via confirmatory factor analysis (CFA). The scale’s fit and validity were evaluated via the comparative fit index (CFI); the relative chi-square index, quantified as the minimum discrepancy of CFA/degrees of freedom (CMIN/DF); the normed fit index (NFI); the goodness-of-fit index (GFI); the Tucker–Lewis index (TLI); and the root mean square error of approximation (RMSEA). For CMIN/DF, values of ≤5 indicated good fit, whereas values < 2 indicated excellent fit.^[39]^ CFI, TLI, NFI, and GFI values > 0.90 were deemed indicative of good fit, and values > 0.95 indicated excellent fit.^[40]^ For the RMSEA, values < 0.08 represented good fit, and values < 0.05 indicated excellent fit.^[41]^

To assess the reliability of the Turkish ASES-8, internal consistency, test–retest reliability and measurement error analyses were conducted. Cronbach’s α was calculated for the scale, with an α value above 0.70 considered reliable.^[42]^ To evaluate test–retest reliability, the intraclass correlation coefficient (ICC) was calculated for the total ASES-8 score, using a 2-way random-effects model with absolute agreement. ICC values < 0.4 were considered poor, values between 0.4 and 0.59 were considered moderate, values between 0.6 and 0.74 were considered good, and values ≥ 0.75 were considered excellent.^[39]^ For the individual items, quadratic weighted kappa coefficients were computed to assess agreement between the test and retest scores, as item responses are ordinal. Kappa values were interpreted as follows: values below 0.20 were considered poor, values between 0.21 and 0.40 as fair, values between 0.41 and 0.60 as moderate, values between 0.61 and 0.80 as good, and values between 0.81 and 1.00 as very good agreement.^[43]^ For measurement error, standard error of the measurement (SEMagreement) and smallest detectable change (SDC) at the individual-level were used. SEMagreement was estimated by the formula [differenceSD/√2], in which differenceSD was the standard deviation (SD) of the difference between the test and retest score. SDC was estimated by [SEM*1.96*√2]. In addition, the percentage of absolute agreement was calculated.

## 3. Results

A total of 80 patients with knee osteoarthritis (KOA) participated in the study. The participants’ mean age was 59.7 ± 4.2 years (range: 26–92), and the majority were female (83.8%). The median disease duration was 7 years (IQR: 11). Of the participants, 31.3% were smokers, 40% were literate, and 17.5% had a university degree. Treatments received included platelet-rich plasma (32.5%), physical therapy (48.8%), and hyaluronic acid injections (32.5%). Detailed demographic and clinical characteristics are presented in Table 1.

**Table 1:** Sociodemographic characteristics and clinical findings of the patients.

Characteristic	All (N:80)
Gender, n (%)	
Female	67 (83.8)
Male	13 (16.2)
Age, yr, mean (SD)	59.7 (14.2)
Height, cm, median (IQR)	161 (9)
Weight, kg, median (IQR)	77 (16)
BMI, median (IQR)	28.7 (7.98)
Education level, n (%)	
Illiterate	4 (5)
Literate with no formal schooling	32 (40)
Primary school	11 (13.8)
Middle school	7 (8.8)
High school	5 (6.3)
University	14 (17.5)
Master’s degree	7 (8.8)
Smoker, n (%)	25 (31.3)
Duration of disease, yr, median (IQR)	7 (11)
PRP received, n (%)	26 (32.5)
Physical therapy received, n (%)	39 (48.8)
Hyaluronic acid received, n (%)	26 (32.5)
Other treatments received, n (%)	4 (5)
ASES-8, mean, range 1–10 (SD)	4.33 (2.25)
DASS-42 depression, mean (SD)	10.7 (6.2)
DASS-42 anxiety, mean (SD)	15.4 (9.38)
DASS-42 stress, median (IQR)	19 (23)
TSK, median (IQR)	49.5 (29)
PCS, median (IQR)	40 (21)
PSEQ, median (IQR)	26.5 (31)
WOMAC, median (IQR)	76 (54)

Participants with low literacy or illiteracy completed the questionnaires through a standardized read-aloud procedure conducted by the researchers, ensuring impartiality and consistency.

ASES-8 = arthritis self-efficacy 8 item, BMI = body mass index, DASS-42, depression anxiety stress scales 42 item, IQR = interquartile range, PCS = pain catastrophizing scale, PRP = platelet reach plasma, PSEQ = pain self-efficacy questionnaire, SD = standard deviation, TSK = Tampa scale of kinesiophobia, WOMAC = Western Ontario and Mcmaster Universities Osteoarthritis Index.

All participants completed the Turkish ASES-8 and the other administered scales in full, resulting in a 100% completion rate. No missing responses were recorded for any item.

No content from the original English version of the ASES-8 was identified as culturally inappropriate or conceptually inconsistent for Turkish patients with KOA. Therefore, no cultural modifications were deemed necessary. Based on feedback from 10 diverse patients during cognitive debriefing interviews, the Turkish version of the ASES-8 was found to be clear, comprehensible, and easy to complete. All participants completed the scale without difficulty, indicating good face validity and acceptability. There were no missing responses for the Turkish ASES-8 or the other administered scales. The mean per-item ASES-8 score was 4.33 ± 2.25 (range: 1–10).

The distribution of responses for each item and the total score approximated a normal distribution. Detailed distributional characteristics and centering are presented in Table 2. Floor effects were observed in item 2 (15%), item 3 (15%), item 4 (16.3%), and item 6 (17.5%), where more than 15% of respondents selected the lowest possible score. There were no ceiling or floor effects for the total score.

**Table 2:** Distribution characteristics, reliability indices (ICC/kappa), and absolute agreement for item and scale scores of the Turkish ASES-8.*

	Mean (SD)	Skewness (SE), *z*-score	Kurtosis (SE), *z*-score	Kappa†/ICC‡ (CI)	% Absolute agreement	SEM	SDC
Item 1	4.43 (2.15)	0.352 (0.269), 1.309	−0.330 (0.532), −0.620	0.992† (0.986 –0.998)	92.5	0.194	0.537
Item 2	4.31 (2.55)	0.432 (0.269), 1.606	−0.722 (0.532), −1.357	0.995† (0.990 –0.999)	93.8	0.225	0.623
Item 3	4.09 (2.29)	0.470 (0.269), 1.747	−0.614 (0.532), −1.154	0.995† (0.990–1.0)	95.0	0.158	0.438
Item 4	4.36 (2.62)	0.579 (0.269), 2.153	−0.510 (0.532), −0.958	0.999† (0.997–1.0)	98.8	0.079	0.219
Item 5	4.33 (2.49)	0.407 (0.269), 1.514	−0.857 (0.532), −1.611	0.998† (0.995–1.0)	97.5	0.111	0.307
Item 6	4.38 (2.70)	0.482 (0.269), 1.792	−0.897 (0.532), −1.685	0.998† (0.996–1.0)	97.5	0.112	0.311
Item 7	4.44 (2.55)	0.551 (0.269), 2.048	−0.719 (0.532), −1.351	0.998† (0.995–1.0)	97.5	0.112	0.311
Item 8	4.35 (2.49)	0.566 (0.269), 2.104	−0.653 (0.532), −1.227	1† (1.0–1.0)	100	0	0
ASES score	4.33 (2.25)	0.501 (0.269), 1.863	−0.724 (0.532), −1.361	0.966‡ (0.947 –0.978)	80.0	0.417	1.158

CI = confidence interval, ICC = intraclass correlation coefficient, SD = standard deviation, SDC = smallest detectable change, SE = standard error, SEM = standard error of measurement.

* All reliability coefficients are statistically significant at *P* < .001.

† Quadratic weighted kappa coefficient.

‡ ICC coefficient.

Predefined hypotheses regarding the expected relationships between ASES-8 scores and comparator instruments were confirmed. As hypothesized, the Turkish ASES-8 showed a strong positive correlation with the PSEQ (*r* = 0.912, *P* < .001). Additionally, strong negative correlations were observed with the depression (*r* = –0.798), anxiety (*r* = –0.838), and stress (*r* = –0.862) subscales of the DASS-42; the Tampascale for kinesiophobia (*r* = –0.917); the pain catastrophizing scale (*r* = –0.897); and the WOMAC total score (*r* = –0.917), supporting the construct validity of the Turkish ASES-8 (Table 3).

**Table 3:** Correlation of the ASES-8 score with other scales (N = 80).

	PSEQ	DASS-42 depression subscale	DASS-42 anxiety subscale	DASS-42 stress subscale	TSK	PCS	WOMAC
ASES	0.912*	−0.798†	−0.839†	−0.862*	−0.917*	−0.897*	−0.917*

All values are statistically significant, *P* ˂ 0.001.

ASES-8 = arthritis self-efficacy scale-8 item, DASS-42 = depression anxiety stress scales 42 item, PCS = pain catastrophizing scale, PSEQ = pain self-efficacy questionnaire, TSK = Tampa scale of kinesiophobia, WOMAC = Western Ontario and Mcmaster Universities Osteoarthritis Index.

* Spearman’s rho.

† Pearson’s correlation coefficient.

**Figure 1.**
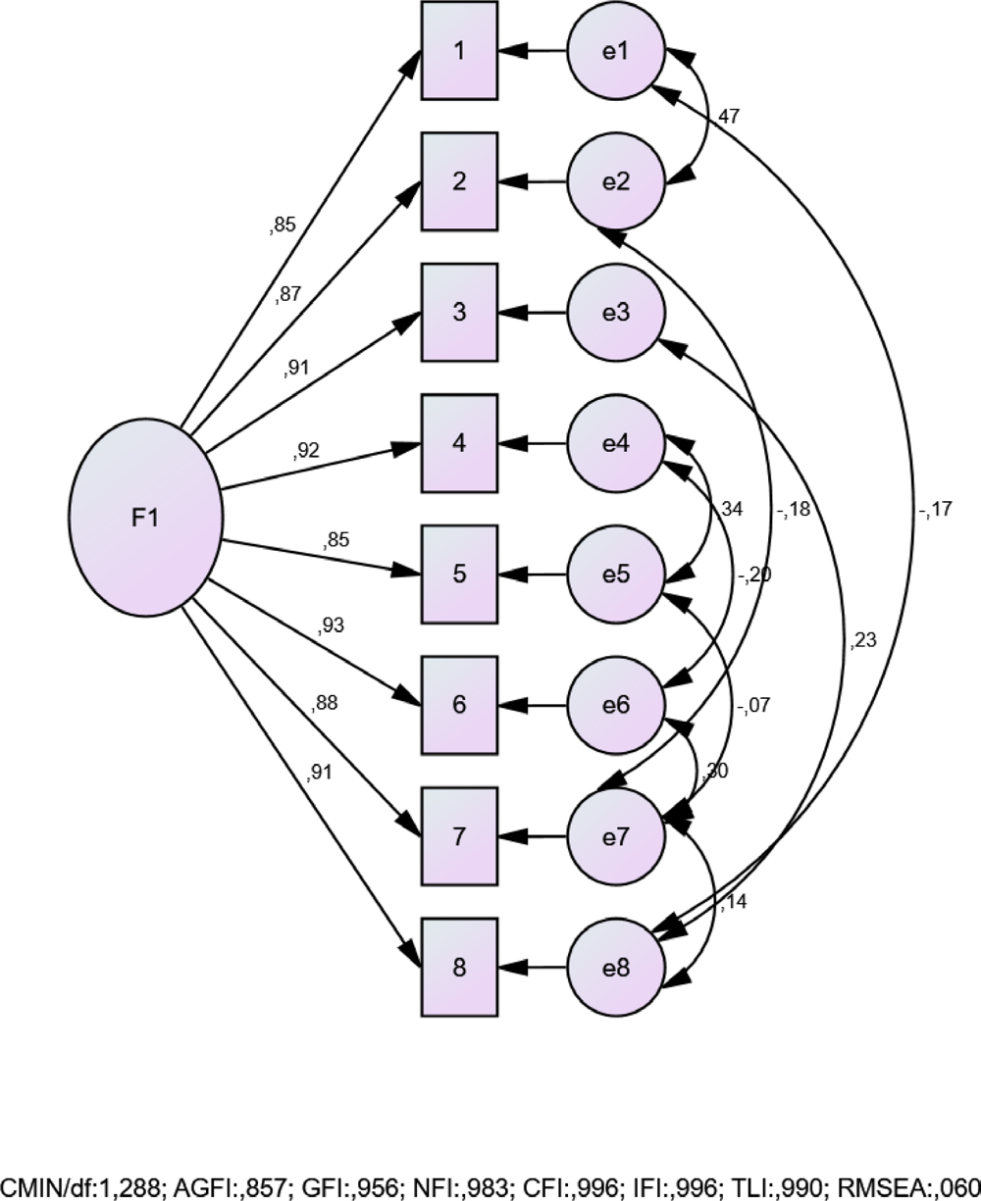
Confirmatory factor analysis (CFA) of the Turkish version of the arthritis self-efficacy scale-8 (ASES-8). The 1-factor structure showed good to excellent fit indices (CMIN/DF: 1.288, CFI: 0.966, TLI: 0.990, GFI: 0.956, NFI: 0.983, RMSEA: 0.060), confirming the unidimensional model. ASES-8 = arthritis self-efficacy scale-8, CFA = confirmatory factor analysis, CMIN/DF = the minimum discrepancy of CFA/degrees of freedom, GFI = goodness-of-fit index, NFI = normed fit index, RMSEA = root mean square error of approximation, TLI = Tucker–Lewis index.

CFA confirmed the unidimensional structure of the ASES-8, in line with its original theoretical model. The fit indices indicated acceptable to excellent model fit: [CMIN/DF: 1.288, CFI: 0.966, TLI: 0.990, GFI: 0.956, NFI: 0.983, RMSEA: 0.060]. A visual representation of the model is shown in Figure 1.

The Turkish ASES-8 demonstrated excellent internal consistency, with a Cronbach’s α of 0.968. The ICC for the total score was 0.966, indicating excellent agreement. At the item-level, quadratic weighted kappa coefficients ranged from 0.992 to 1.000, reflecting very good agreement. Standard error of measurement (SEM) values for individual items ranged from 0.079 (item 4) to 0.225 (item 2), indicating low measurement error across items. Corresponding SDC values ranged from 0.219 to 0.623, representing the minimum detectable change beyond measurement error for each item. The total ASES score had a higher SEM of 0.417 and an SDC of 1.158, reflecting the expected accumulation of measurement variability across all items. These results suggest that while item-level measurement error is minimal, a change of approximately ≥1.16 points is required in the total ASES-8 score to reflect a true change in self-efficacy with 95% confidence In addition, the percentage of absolute agreement for individual items ranged from 92.5% to 100%, further supporting the stability of responses over the 1-week interval (Table 2).

## 4. Discussion

The translation and cross-cultural adaptation of the ASES-8 for Turkish patients with KOA was successfully completed. Linguistic clarity, conceptual equivalence, and cultural relevance were established through forward-backward translation, expert review, and cognitive debriefing interviews. Feedback from 10 patients during pilot testing confirmed that the Turkish version was easy to understand and culturally appropriate. These findings are consistent with the results reported for the Spanish,^[18]^ German,^[17]^ Chinese,^[15]^ Japanese^[20]^, and Arabic^[19]^ versions of the ASES-8. These findings indicate that the scale is suitable for assessing arthritis-related self-efficacy across different cultural contexts.

The Turkish ASES-8 demonstrated excellent psychometric properties. The Turkish ASES-8 demonstrated excellent internal consistency and test–retest reliability, with very high agreement at the item-level. These results support the stability and precision of the scale.

CFA supported the unidimensional structure with good to excellent model fit, consistent with the theoretical construct of the original scale.^[12]^

Construct validity was supported through hypothesis testing, consistent with COSMIN^[23]^ guidelines. Construct validity was supported through strong positive correlations with pain self-efficacy and strong negative correlations with psychological distress (depression, anxiety, stress; DASS-42), kinesiophobia (TSK), pain catastrophizing (PCS), and disability WOMAC), consistent with theoretical expectations. These findings support the theoretical assumptions that higher self-efficacy is associated with better coping, lower distress, and improved function in KOA patients.^[44–47]^

Although moderate correlations with psychological symptoms were expected, the observed associations were stronger than anticipated. This discrepancy may reflect specific psychosocial characteristics of the Turkish population, where low self-efficacy may be more strongly linked to symptoms of depression, anxiety, and stress. It is also possible that Turkish individuals place greater emphasis on confidence in managing illness, making this construct more closely intertwined with mental well-being.^[48]^ These findings suggest that the ASES-8 may serve as a particularly sensitive indicator of psychological distress in Turkish patients with KOA.

The magnitude and direction of these correlations have practical implications. For example, the strong negative correlation with the WOMAC suggests that the ASES-8 could be used to identify patients at risk for functional limitations, while the association with PSEQ indicates strong convergent validity. These relationships reinforce the scale’s relevance in rehabilitation planning, patient education, and psychological support interventions.^[1,12]^

Interpretability was addressed through an analysis of response distributions. No ceiling effects were observed, but 4 items (items 2–4 and 6) showed floor effects exceeding 15%. These findings partially align with previous reports noting end-point clustering in 0 to 10 numeric rating scales.^[12]^ Notably, the floor effects in items 2, 4, and 6 may reflect difficulties in comprehension or applicability due to discrepancies between the item content and the daily life practices of the Turkish population. These items may not fully align with local cultural norms or daily routines, possibly resulting in misinterpretation or non-applicability, leading to low scores. This suggests a potential area for refinement in future versions of the instrument to enhance cultural congruence. However, the total score showed no floor or ceiling effects, and item distributions were approximately normal. Despite the presence of item-level floor effects, the absence of floor or ceiling effects at the total score level indicates that the Turkish ASES-8 retains adequate sensitivity and inclusiveness for a broad KOA population. This suggests its suitability for both clinical and research use across varied demographic and treatment backgrounds. Future studies should investigate these response tendencies in larger and more diverse Turkish populations.

Compared to previous validation studies of the ASES-8,^[12,17–20]^ the Turkish version demonstrated stronger psychometric performance, suggesting enhanced construct clarity and contextual alignment within the Turkish KOA population. Despite these advantages, the presence of item-level floor effects – unlike in previous validations – highlights the importance of further cultural adaptation for certain items. Moreover, our study expanded upon earlier validations by incorporating advanced psychometric evaluations such as measurement error analysis, interpretability indices, and absolute agreement metrics, further supporting the Turkish ASES-8 as a robust and clinically applicable tool for both national and international research contexts.

Some measurement properties, such as responsiveness to change, minimal important change, and longitudinal validity, were not assessed in this study. These aspects are essential for evaluating clinical utility in monitoring treatment outcomes and should be addressed in future longitudinal research.

The availability of a validated Turkish version of the ASES-8 provides a reliable and culturally appropriate tool for assessing arthritis-related self-efficacy in clinical and research settings. Clinicians can use it to identify patients needing targeted interventions, monitor progress over time, and personalize rehabilitation strategies. In research contexts, the ASES-8 can serve as an outcome measure in trials of physical therapy, education programs, or psychological interventions.^[20]^ Its cross-cultural comparability enhances its utility in multinational studies and meta-analyses of self-efficacy in musculoskeletal conditions.^[17–20]^

## 5. Conclusion

To the best of our knowledge, this is the first study to adapt and validate the ASES-8 for Turkish-speaking patients with knee osteoarthritis. Following COSMIN guidelines, we conducted a comprehensive psychometric evaluation covering structural validity, internal consistency, test–retest reliability, measurement error, interpretability, and construct validity through hypothesis testing. The Turkish version of the ASES-8 demonstrated excellent reliability and validity, with no ceiling effects and only limited item-level floor effects. The absence of missing responses and the scale’s acceptability suggest that it is a feasible and inclusive tool for this population. Based on our findings in Turkish patients with KOA, the ASES-8 appears to be a clinically applicable and culturally appropriate instrument for assessing arthritis-related self-efficacy. Future studies are warranted to evaluate responsiveness, minimal important change, and longitudinal utility in broader and more diverse patient populations.

## Acknowledgments

We would like to thank Enago (www.enago.com) for the English language review.

## Author contributions

**Conceptualization:** Mehmet Serkan Kiliçoğlu, Delal Öztürk, Ozan Volkan Yurdakul.

**Data curation:** Mehmet Serkan Kiliçoğlu, Buğra İnce.

**Formal analysis:** Mehmet Serkan Kiliçoğlu, Delal Öztürk, Buğra İnce, Ozan Volkan Yurdakul.

**Investigation:** Mehmet Serkan Kiliçoğlu, Delal Öztürk, Teoman Aydin.

**Methodology:** Mehmet Serkan Kiliçoğlu, Delal Öztürk, Ozan Volkan Yurdakul, Teoman Aydin.

**Project administration:** Mehmet Serkan Kiliçoğlu.

**Resources:** Mehmet Serkan Kiliçoğlu, Buğra İnce.

**Software:** Mehmet Serkan Kiliçoğlu, Delal Öztürk.

**Supervision:** Mehmet Serkan Kiliçoğlu, Ozan Volkan Yurdakul, Teoman Aydin.

**Validation:** Mehmet Serkan Kiliçoğlu, Buğra İnce, Ozan Volkan Yurdakul.

**Visualization:** Mehmet Serkan Kiliçoğlu, Teoman Aydin.

**Writing – original draft:** Mehmet Serkan Kiliçoğlu, Ozan Volkan Yurdakul.

**Writing – review & editing:** Mehmet Serkan Kiliçoğlu, Buğra İnce, Teoman Aydin.

## Supplementary Material


