# Supplementary material for: Cross-cultural adaptation and validation of the Turkish version of the arthritis self-efficacy scale-8 (ASES-8) in patients with knee osteoarthritis
Source: Medicine (Baltimore). 2025 Nov 7;104(45):e45599. doi: 10.1097/MD.0000000000045599 (PMC12599695; doi:10.1097/MD.0000000000045599)
Supplement: Supplementary file 1 [file medi-104-e45599-s001.pdf]

Supplemental Digital Content 1. Permission letter for the use of the Arthritis Self-Efficacy Scale-8 (ASES-8). (JPEG file)

22:02

PLEASE Professor can you take a look for this mail? regardly. Inbox

me May 31, 2023
to lorig@stanford.edu

Dear Professor Lorig, I am a resident doctor training in a Rheumatology course at Bezmialem University in İstanbul /Turkey. I am interested in translating and adapting the ASES-8 form to Turkish and then searching for the reliability and validity of it. I am writing to you asking if I could take your permission to start the process.
Yours sincerely

Kate R Lorig Jun 1, 2023
to me

These scales are in the public domain and you are free to use them as you wish. We would love a Turkish copy if you translate so we can share with others. Kate

...

---

me Jun 13, 2023
to Kate

Thank you for your kindness dear Professor, then we will share you the turkish copy if we can validate it successfully
Kind regards 😊

Kate R Lorig <~~lorig@stanford.edu~~>, 1 Haz 2023

Supplemental Digital Content 2. Permission letter for the use of the Pain Catastrophizing Scale (PCS). (JPEG file)

10:25 LTE 31

ePROVIDE™: Your User License Agreement - Pain Catastrophizing Scale - 108382

Inbox

noreply 10:18
to me

Dear Delal Ozturk,

Thank you for using the online distribution on ePROVIDE™ for the use of this COA.

Please find attached your completed User License Agreement.

This COA may be under specific conditions of use and copyright ownership.

By accepting the General and Special Terms of this User License Agreement, you have acknowledged that you will respect these conditions, and especially:

- You will only use the COA in the context of use that you have indicated
- You will not modify the COA
- You will not translate the COA without contacting Mapi Research Trust beforehand for possible specific conditions
- You will not distribute the COA to other third parties

We invite you to refer to the attached Terms for [illegible]

Supplemental Digital Content 3. Permission letter for the use of the Pain Self-Efficacy Questionnaire (PSEQ). (JPEG file)

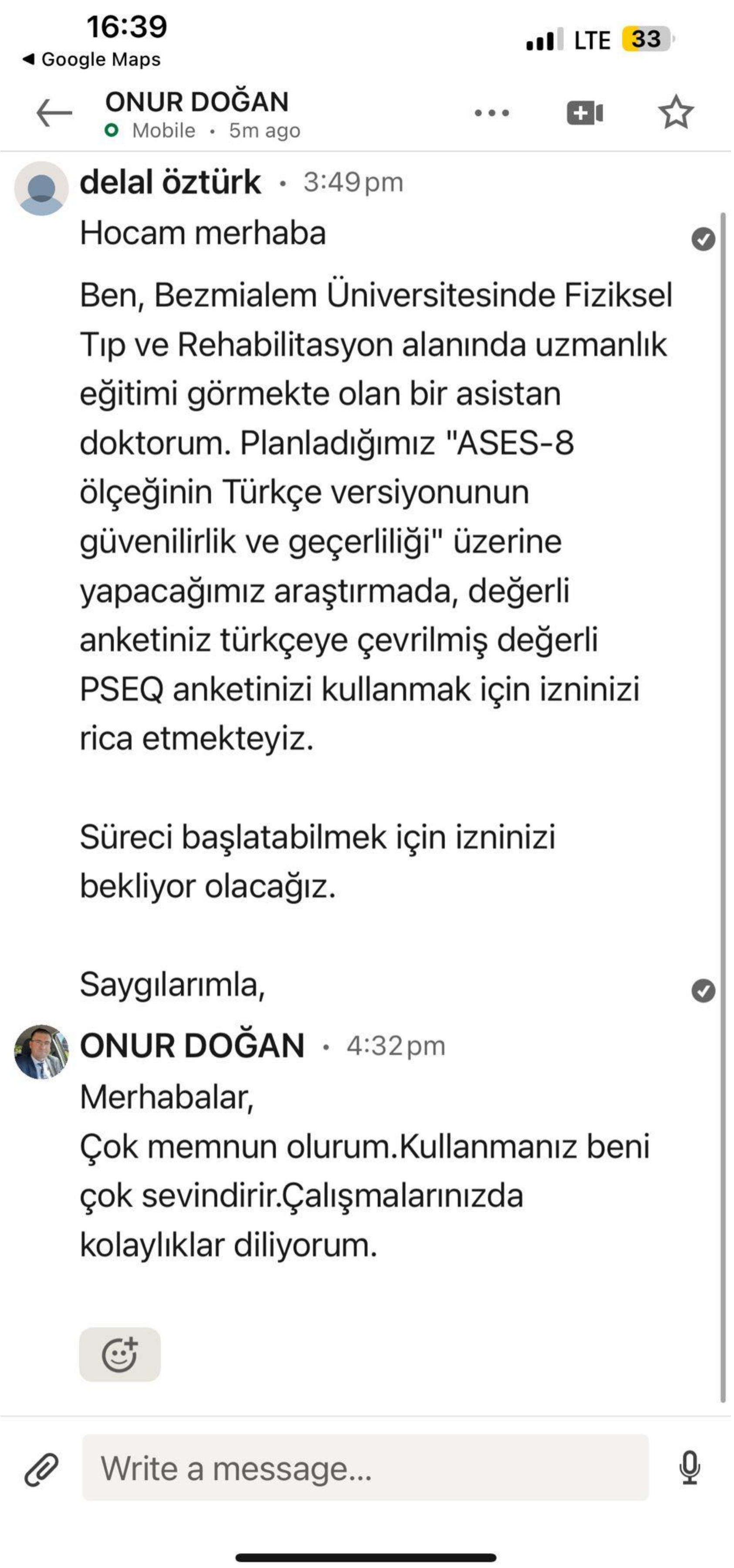
16:39
◂ Google Maps
LTE 33

ONUR DOĞAN
Mobile · 5m ago

delal öztürk · 3:49 pm

Hocam merhaba

Ben, Bezmialem Üniversitesinde Fiziksel Tıp ve Rehabilitasyon alanında uzmanlık eğitimi görmekte olan bir asistan doktorum. Planladığımız "ASES-8 ölçeğinin Türkçe versiyonunun güvenilirlik ve geçerliliği" üzerine yapacağımız araştırmada, değerli anketiniz türkçeye çevrilmiş değerli PSEQ anketinizi kullanmak için izninizi rica etmekteyiz.

Süreci başlatabilmek için izninizi bekliyor olacağız.

Saygılarımla,

ONUR DOĞAN · 4:32 pm

Merhabalar,
Çok memnun olurum.Kullanmanız beni çok sevindirir.Çalışmalarınızda kolaylıklar diliyorum.

Write a message...

Supplemental Digital Content 4. Permission letter for the use of the Tampa Scale of Kinesiophobia (TSK). (JPEG file)

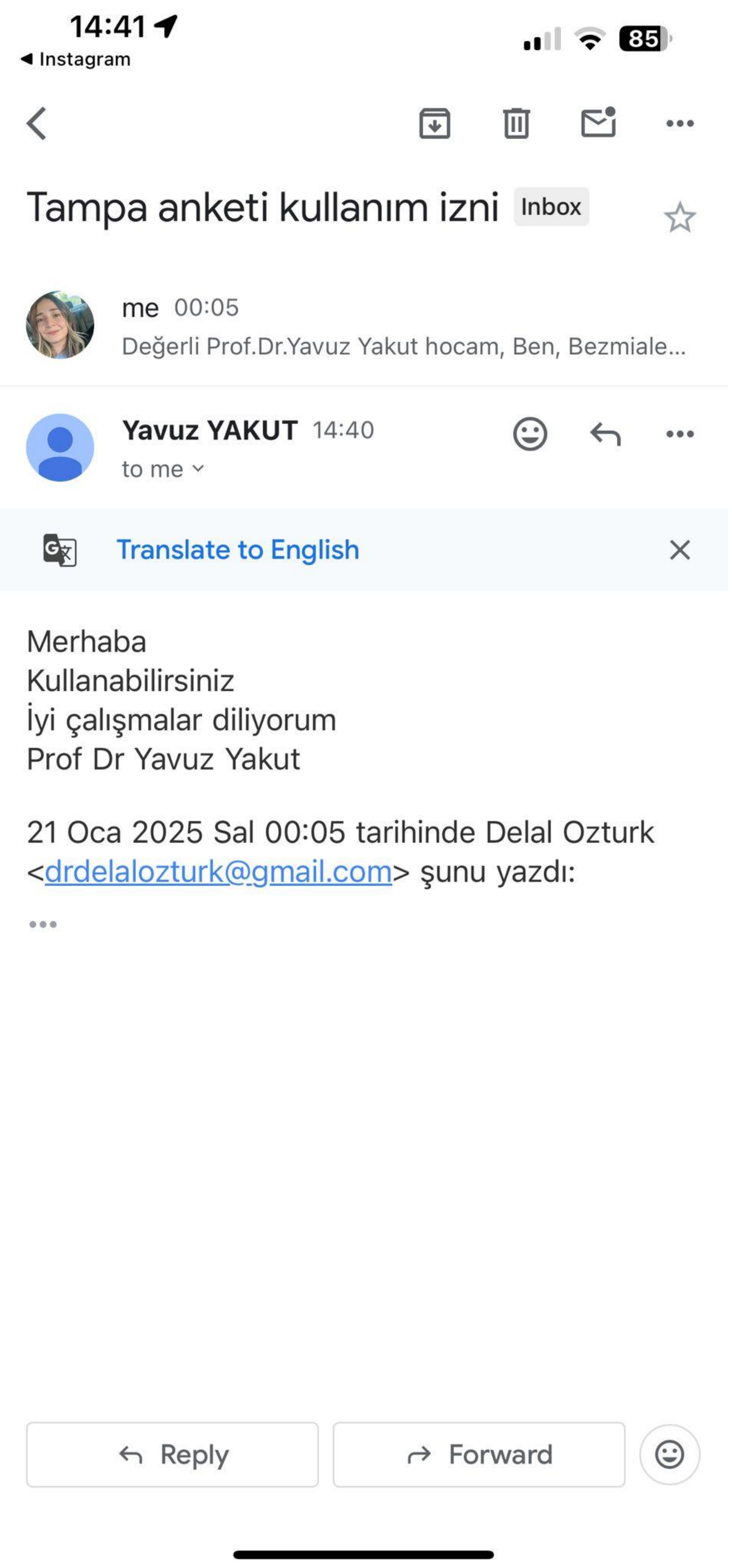
14:41
◀ Instagram
85

Tampa anketi kullanım izni Inbox

me 00:05
Değerli Prof.Dr.Yavuz Yakut hocam, Ben, Bezmiale...

Yavuz YAKUT 14:40
to me

Translate to English

Merhaba
Kullanabilirsiniz
İyi çalışmalar diliyorum
Prof Dr Yavuz Yakut

21 Oca 2025 Sal 00:05 tarihinde Delal Ozturk <drdelalozturk@gmail.com> şunu yazdı:

Reply Forward

Supplemental Digital Content 5. Permission letter for the use of the Western Ontario and McMaster Universities Osteoarthritis Index (WOMAC). (JPEG file)

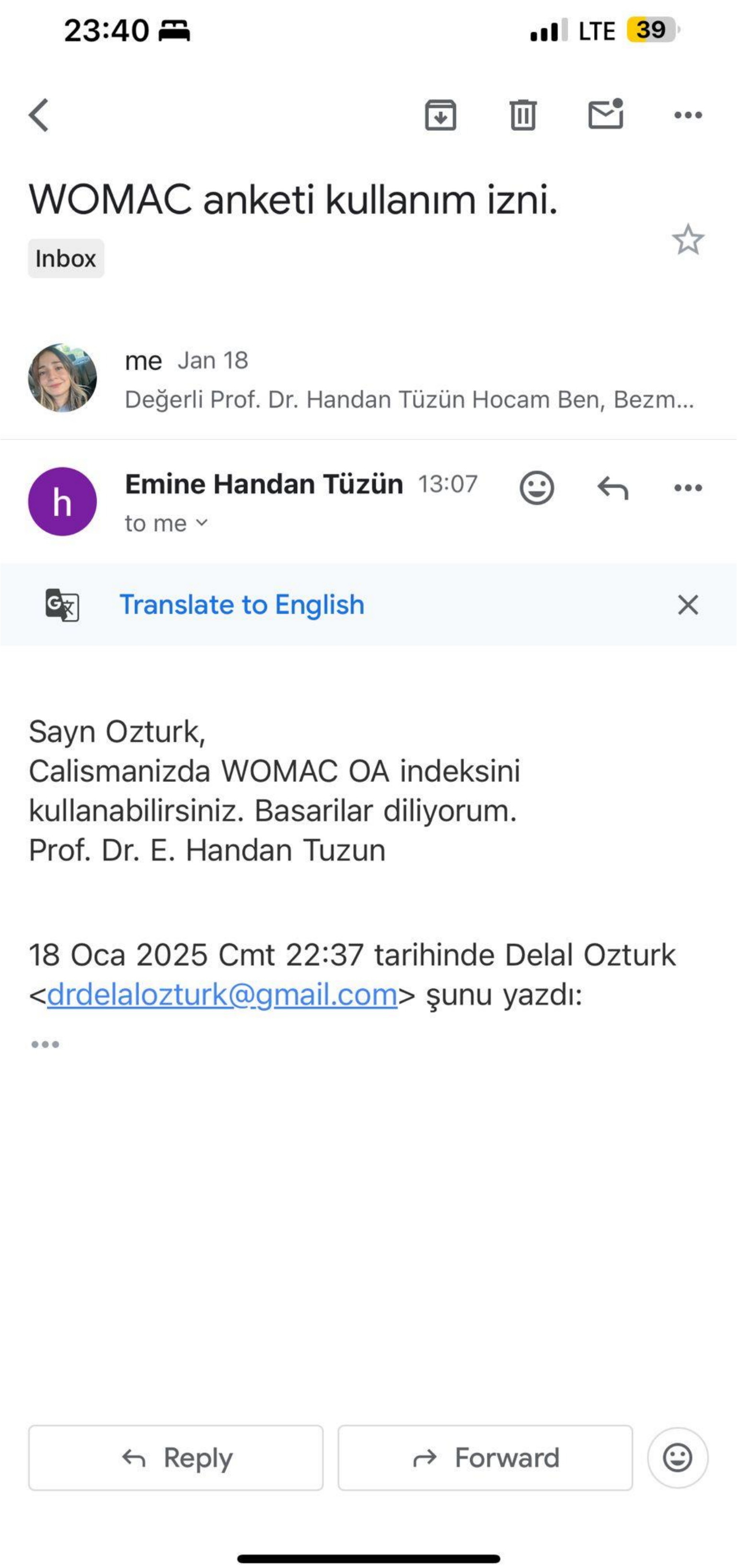

23:40

LTE 39

WOMAC anketi kullanım izni.

Inbox

me Jan 18

Değerli Prof. Dr. Handan Tüzün Hocam Ben, Bezm...

Emine Handan Tüzün 13:07

to me

Translate to English

Sayn Ozturk,
Calismanizda WOMAC OA indeksini kullanabilirsiniz. Basarilar diliyorum.
Prof. Dr. E. Handan Tuzun

18 Oca 2025 Cmt 22:37 tarihinde Delal Ozturk <drdelalozturk@gmail.com> şunu yazdı:

...

Reply

Forward

Document Date and Number: 10.02.2025-182027

REPUBLIC OF TURKEY
BEZMIALEM VAKIF UNIVERSITY RECTOR
Technology Transfer Office

Number : E-54022451-050.04-182027 10.02.2025
Subject : 2023_211 Petition Request in English

Dear Dr. Lecturer. Member of Mehmet Serkan KILIÇOĞLU

Relevance : 2023_211 Petition Request in English

Your application entitled "Cross-cultural adaptation and validation of a Turkish version of the Arthritis Self-Efficacy Scale-8 (ASES-8) for patients with knee osteoarthritis" was evaluated and approved by the Ethics Committee of NonInvasive Studies with a document number of 122617 in September, 15 2023. This acceptance letter is prepared in English because of your request.

Sincerely,

Prof.Dr. Hayrettin ÖZTÜRK
Non-Interventional Research Ethics
Committee Chairman

This document has been legally e-signed.
Verification code :BSAB6ZAZ34 Document Tracking: https://turkiye.gov.tr/ebd?eK=5394&eD=BSAB6ZAZ34&eS=182027
Address:Bezmiâlem Vakıf Üniversitesi Adnan Menderes Bulvarı (Vatan Caddesi)
Fatih / İstanbul
Phone:0 (212) 523 22 88 Fax:0 (212) 533 23 26
e-mail:info@bezmialem.edu.tr URL:www.bezmialem.edu.tr
Detailed Info: Enes KAMACI
Title: Secretary of the Ethics Committee

This document is signed with secure e-signature.

SMRC
Self-Management
Resource Center

Arthritis Self-Efficacy (8-Item)

For each of the following questions, please circle the number that corresponds to how certain you are that you can do the following tasks regularly at the present time.

1. How certain are you that you can decrease your pain quite a bit?

very uncertain	1	2	3	4	5	6	7	8	9	10	very certain

2. How certain are you that you can keep your arthritis or fibromyalgia pain from interfering with your sleep?

very uncertain	1	2	3	4	5	6	7	8	9	10	very certain

3. How certain are you that you can keep your arthritis or fibromyalgia pain from interfering with the things you want to do?

very uncertain	1	2	3	4	5	6	7	8	9	10	very certain

4. How certain are you that you can regulate your activity so as to be active without aggravating your arthritis or fibromyalgia?

very uncertain	1	2	3	4	5	6	7	8	9	10	very certain

5. How certain are you that you can keep the fatigue caused by your arthritis or fibromyalgia from interfering with the things you want to do?

very uncertain	1	2	3	4	5	6	7	8	9	10	very certain

6. How certain are you that you can do something to help yourself feel better if you are feeling blue?

very uncertain	1	2	3	4	5	6	7	8	9	10	very certain

7. As compared with other people with arthritis or fibromyalgia like yours, how certain are you that you can manage pain during your daily activities?

very uncertain	1	2	3	4	5	6	7	8	9	10	very certain

8. How certain are you that you can deal with the frustration of arthritis or fibromyalgia?

very uncertain	1	2	3	4	5	6	7	8	9	10	very certain

Scoring

The score for each item is the number circled. If two consecutive numbers are circled, code the lower number (less self-efficacy). If the numbers are not consecutive, do not score the item. The score for the scale is the mean of the eight items. If more than two items are missing, do not score the scale.

This scale is free to use without permission

Characteristics

Tested on 175 subjects with arthritis.

No. of items	Observed Range	Mean	Standard Deviation	Internal Consistency Reliability	Test-Retest Reliability
8	1-10	5.53	2.20	.92	NA

Source of Psychometric Data

Stanford Arthritis Self-Management Study participants. Unpublished.

Comments

This is the scale we used in our most recent studies, as it is much less burdensome for subjects than the original 3 scales with 20 total items. We did not included function items because we also used the HAQ and there is a high correlation between the SE function scale and the HAQ disability scale. This scale is available in Spanish.

References

Lorig K, Chastain RL, Ung E, Shoor S, & Holman HR: Development and evaluation of a scale to measure self-efficacy in people with arthritis. *Arthritis and Rheumatism*, 32, 1, 1989, pp. 37-44 (original scales).

This scale is free to use without permission

Self-Management Resource Center
smrc@selfmanagementresource.com
https://selfmanagementresource.com

ASES-8 Türkçe:

1. Ağrını az da olsa azaltabileceğinden ne kadar eminsin?

2. Eklem ağrıların uykunu bölmesinden alıkoyabileceğinizden ne kadar eminsin ?

3. Eklem ağrılarını istediklerini yapmaktan engellemesini ne kadar kontrol edebileceğinden eminsin ?

4. Aktivitelerini ağrını arttırmayacak şekilde düzenleyip aktif kalabileceğinden ne kadar eminsin?

5. Eklem ağrılarının oluşturduğu yorgunluk istediklerini yapmaktan engellemesini ne kadar kontrol edebileceğinden eminsin ?

6. Eğer üzgün hissediyorsanız kendinizi daha iyi hissetmenize yardımcı olabileceğinizden ne kadar eminsiniz?

7. Kendinizi sizin gibi eklem ağrıları deneyimleyen insanlarla karşılaştırdığınızda, eklem ağrılarınızı günlük hayatınızda kontrol edebileceğinizden ne kadar eminsiniz?

8. Hastalığın verdiği hüsran ile başa çıkabileceğinizden ne kadar eminsiniz?
